# Supplementary material for: A Modified Basophil Activation Test for the Clinical Management of Immediate Hypersensitivity Reactions to Paclitaxel: A Proof-of-Concept Study
Source: Cancers (Basel). 2023 Dec 13;15(24):5818. doi: 10.3390/cancers15245818 (PMC10741873; doi:10.3390/cancers15245818)
Supplement: Supplementary file 1 [file cancers-15-05818-s001.zip › cancers-2725983-supplementary.pdf]

SUPPLEMENTARY MATERIALS

Supplementary Methods

iHSR classification

iHSR grade was set into three groups, according to published severity grading system [Sref 1]:

- grade-1 (mild reactions): symptoms and signs limited to skin (erythema, urticaria, angioedema);
- grade-2 (moderate reactions): involvement of respiratory, cardiovascular or gastrointestinal systems (dyspnea, stridor, wheeze, nausea, vomiting, abdominal pain, dizziness, diaphoresis, chest or throat tightness);
- grade-3 (severe reactions): hypoxemia (cyanosis or $SpO_2 \leq 92\%$), hypotension (systolic blood pressure<90mmHg in adults), collapse, loss of consciousness, incontinence.

Skin testing

Skin tests (ST) were performed after at least 3 weeks and not later than 6 months from iHSR episodes [Sref 1]. Skin prick test (SPT) was performed by skin pricking in the forearm volar surface with a lancet through a drop of culprit drug (6mg/ml paclitaxel and 10mg/ml carboplatin). In case of negative results, intradermal ST (IDST) was performed starting from the lowest drug concentration (0.1 and 1mg/ml paclitaxel and 1, 5 and 10mg/ml carboplatin) [Sref 2]. SPT and IDST were read as previously described [Sref 1]. A positive reaction was defined as a wheal with a diameter at least 3 mm larger than that produced by a negative control (dilutor). Histamine (10mg/ml) was used as positive control. Positive ST reactions are suggestive for IgE-mediated reactions. Negative ST results are suggestive for non-immune reactions [Sref 2].

Blood Samples

Peripheral blood samples (3ml) were collected by venipuncture into an EDTA-containing vacutainer blood collection tube.

Chemotherapeutic agents

Paclitaxel (0.6mg/ml) and carboplatin (1mg/ml) were obtained from infusion solution bags within 4 hours from their preparation. Serial dilutions of each drug were tested in the BAT by diluting original infusion solutions in phosphate buffered saline (PBS, OXOID, Hampshire, UK).

Supplementary Results

Table S1 Monoclonal antibodies used in the BAT.

Purpose	Clone	Specificity	Fluorochrome	Producer
Basophil identification	5E8	Anti-CCR3	APC	BioLegend (San Diego, CA, USA)
	BM16	Anti-CRTH2	FITC	Beckman Coulter (Brea, CA, USA)
	J33	Anti-CD45	APC-Alexa750	Beckman Coulter
Activation markers	H5C6	Anti-CD63*	BV421	BioLegend
	97A6	Anti-CD203c	PE	Beckman Coulter
Dumping channel	UCHT1	Anti-CD3	KO	Beckman Coulter

*The anti-CD63 mAb served also to verify basophil responsiveness.

Figure S1. Study flow diagram. iHSR: immediate hypersensitivity reaction. BAT: Basophil Activation Test. ST: Skin Testing.

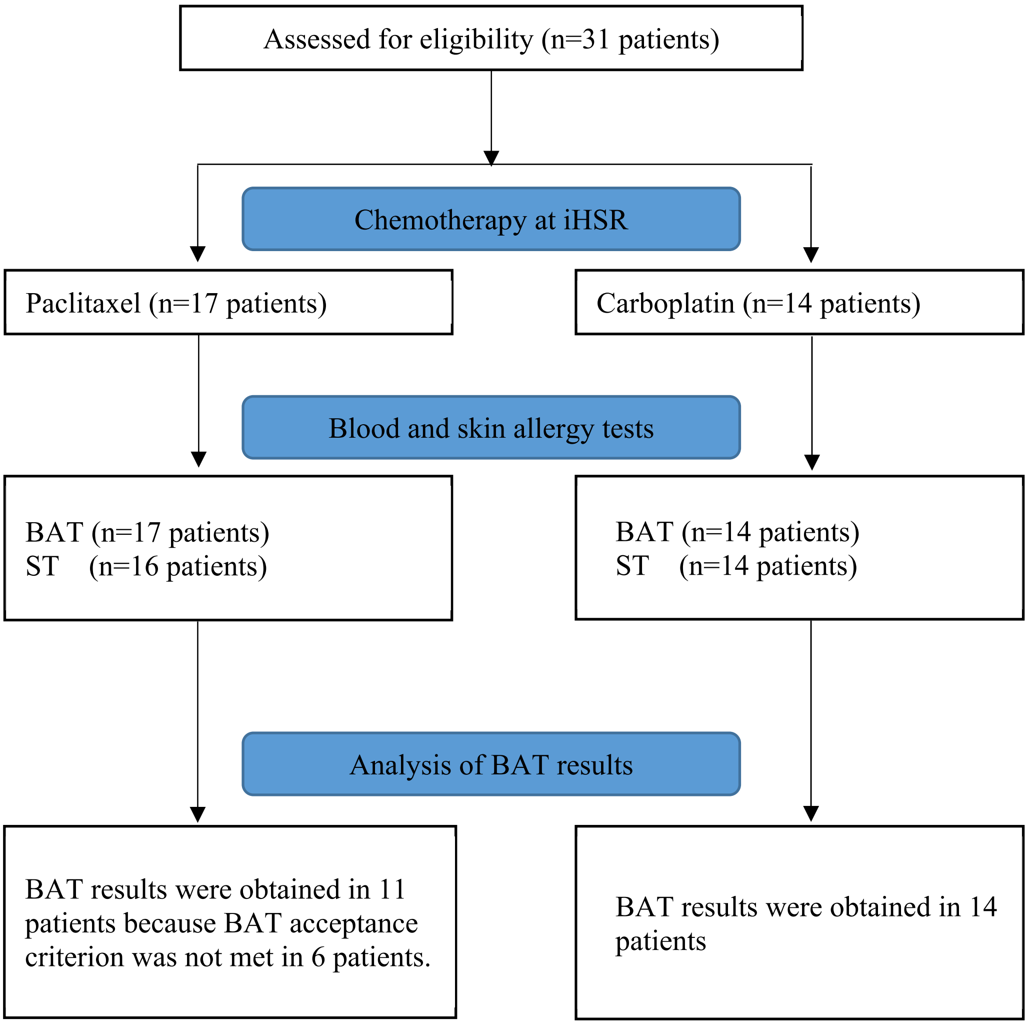

Table S2 Clinical and allergological characteristics of the study population **.**

Characteristics	n (%)
Number of patients	31
Primary tumor	
Breast cancer	4 (12.9)
Ovarian cancer	23 (74.2)
Endometrial cancer	4 (12.9)
Median age (min-max), years	
Chemotherapy at iHSR	
Paclitaxel	17 (54.8)
Carboplatin	14 (45.2)
iHSR Grading§	
iHSR to paclitaxel	
Grade 1	3 (17.6*)
Grade 2	12 (70.6*)
Grade 3	2 (11.8*)
iHSR to carboplatin	
Grade 1	3 (21.4**)
Grade 2	7 (50.0**)
Grade 3	4 (28.6**)
Skin testing	
iHSR to paclitaxel	16
IgE-mediated	2 (12.5)
Non-immune	14 (87.5)
iHSR to carboplatin	14
IgE-mediated	13 (92.9***)
Non-immune	1 (7.1***)

Results are presented as n (%), except where indicated. iHSR: immediate hypersensitivity reaction. *: percentage calculated within patients presenting with iHSR to paclitaxel. **: percentage calculated within patients presenting with iHSR to carboplatin. ***: percentage calculated within patients who performed ST to the culprit drug. §Grading calculated according to Picard [Sref 1]

Figure S2. Effect of paclitaxel on CD63 and CD203c expression in basophils in the BAT.

Basophils from one patient presenting with immediate hypersensitivity reaction to paclitaxel were exposed to paclitaxel at 0 (**A**), 1.2 (**B**), 0.12 (**C**), 0.06 (**D**) and 0.03 (**E**) µg/ml at +37°C for 20 minutes in the BAT. Bivariate histograms show CD203c (x axis) and CD63 (y axis) expression on gated basophils. The paclitaxel-induced basophil degranulation was calculated as increase in the percentage of $CD63^+$ and $CD203c^+$ basophils and in the mean fluorescence intensity (MFI) of CD63 and CD203c. High paclitaxel concentrations (**B**,**C**) induced an unexpected down-modulation of CD63 and, to a lower extent, CD203c on basophils as compared to the control (**A**). No basophil degranulation could be observed at these paclitaxel concentrations. Lower paclitaxel concentrations (**D**,**E**) did not induce CD63 and CD203c down-modulation and paclitaxel-induced basophil degranulation could be observed.

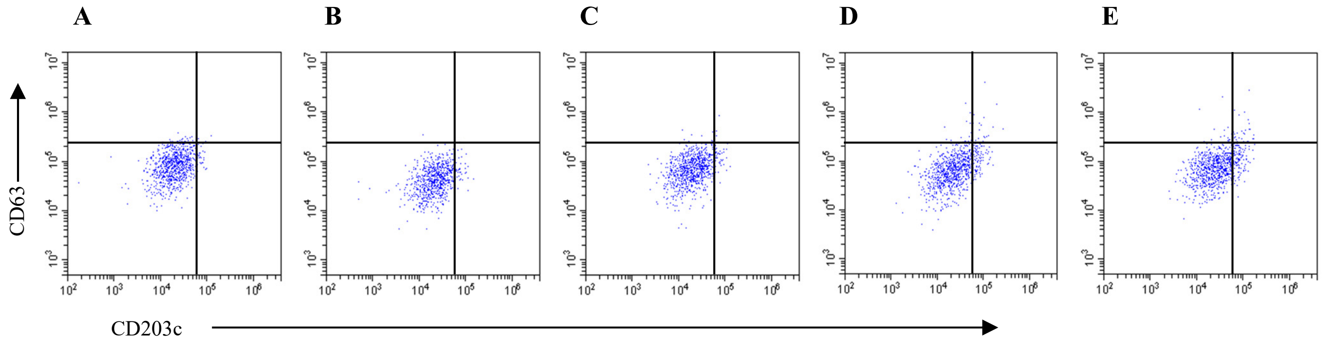

Table S3. Effect of paclitaxel on CD63 and CD203c expression in basophils in the BAT.

The paclitaxel-induced basophil degranulation was calculated as increase in the percentage of $CD63^+$ and $CD203c^+$ basophils and in the mean fluorescence intensity (MFI) of CD63 and CD203c (as shown in the bivariate histograms in Figure S2). High paclitaxel-induced basophil degranulation could be observed only on the condition that only modest or no CD63MFI down-modulation was produced by paclitaxel (paclitaxel 0.06-0.03µg/ml) as compared to the control. *Δ:* change in the specified parameter calculated by subtracting from that value that observed in the control condition (i.e., paclitaxel 0µg/ml).

Degranulation biomarker	Paclitaxel (µg/ml)				
	0	1.2 (Δ)	0.12 (Δ)	0.06 (Δ)	0.03 (Δ)
$CD63^+$ %	1.06	0.15 *(-0.89)*	1.07 *(+0.01)*	2.54 *(+1.48)*	2.86 *(+1.8)*
CD63MFI	102339	50885 *(-51454)*	90418 *(-11921)*	101300 *(-1039)*	107482 *(+5143)*
$CD203c^+$ %	3.53	4.25 *(+0.72)*	6.82 *(+3.29)*	13.7 *(+10.17)*	18.9 *(+15.37)*
CD203cMFI	24063	25083 *(+1020)*	24974 *(+911)*	30692 *(+6629)*	35005 *(+10942)*

Figure S3. SI-CD63%+SI-CD203cMFI levels according to severity grading of the immediate hypersensitivity reaction (iHSR) to drugs. Results are reported for patients presenting with iHSR to paclitaxel (★) and patients presenting with iHSR to carboplatin (★). Horizontal lines represent median values.

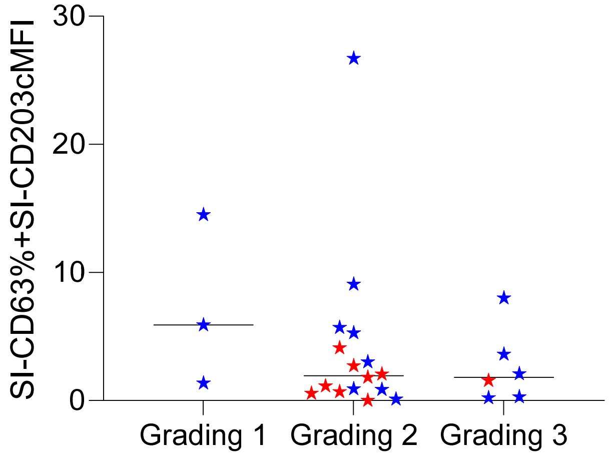

Table S4 Contingency table for skin test (ST) and basophil activation test (BAT) results in patients presenting with immediate hypersensitivity reaction to paclitaxel.

		BAT results		Total
		Negative	Positive	
ST results	Negative	1	7	8
	Positive	0	2	2
	Total	1	9	10

Table S5 Contingency table for skin test (ST) and basophil activation test (BAT) results in patients presenting with immediate hypersensitivity reaction to carboplatin.

		BAT results		Total
		Negative	Positive	
ST results	Negative	0	1	1
	Positive	3	10	13
	Total	3	11	14

Supplementary Data

Assessment of mechanisms involved in in vitro basophil activation in the BAT

Bruton tyrosin kinase (BTK) signaling is critical for basophil degranulation, when induced *via* FcεRI cross-linking [Sref 3-4]. Ibrutinib (IMBRUVICA®, Pharmacyclics LLC, Sunnyvale, CA, USA, and Janssen-Biotech, Inc. Titusville, NJ, USA), is reported to be a selective BTK inhibitor [Sref 3-4] and it has been employed *in vitro* to verify whether basophil degranulation occurs via FcεRI cross-linking, as measured by changes in CD63 expression [Sref 3-4]. Here we used ibrutinib to define mechanisms involved in the basophil degranulation in the BAT of iHSR-Tax^{pos} patients presenting with negative and positive ST. Ibrutinib inhibited paclitaxel-induced CD63 expression in iHSR-Tax^{pos} patients, irrespective of whether patients were ST positive or negative, as well as carboplatin-induced CD63 expression in iHSR-Pl^{pos} patients (Figure S4), implying that in all cases basophil degranulation occurred *via* FcεRI cross-linking [Sref 3-4]. Ibrutinib selectivity for BTK, however, was questioned by experiments showing that ibrutinib inhibited not only the anti-FcεRI mAb-induced basophil degranulation, but in some cases also the BTK-independent fMLP-induced basophil degranulation (Figure S4), suggesting ibrutinib should interfere with pathways other than those involved in the FcεRI cross-linking. The poor selectivity of ibrutinib for BTK we hypothesize here is reminiscent of a previous study revealing a measurable binding affinity of ibrutinib to nine other kinases in addition to BTK [Sref 5]. Thus, intervention of mechanisms of paclitaxel-induced basophil degranulation other than those mediated by the FcεRI might be hypothesized.

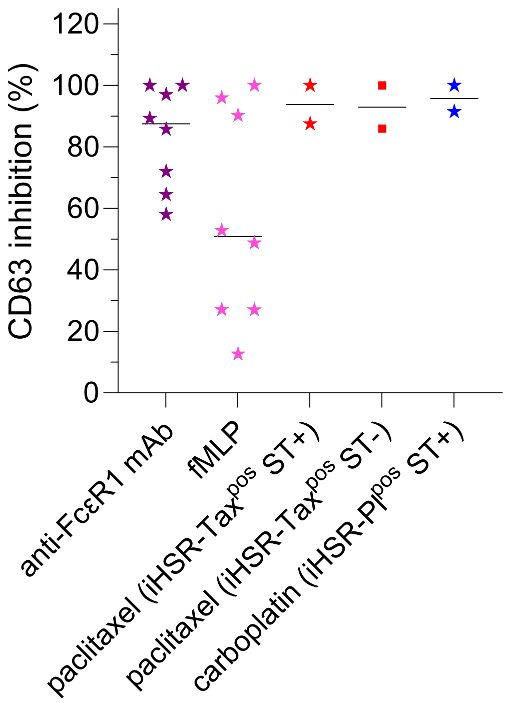

Figure S4. Inhibition of drug-induced basophil activation due to ibrutinib in the BAT. Results are expressed as percent inhibition of CD63 expression induced by ibrutinib 100nM on basophil activation by paclitaxel in patients presenting with immediate hypersensitivity reaction to paclitaxel (iHSR-Tax^{pos}) with positive ★ and negative ■ skin testing (ST), by carboplatin in patients presenting with immediate hypersensitivity reaction to carboplatin (iHSR-Pl^{pos}) with positive ST★, by anti-FcεR1 mAb★ (in iHSR-Tax^{pos} and iHSR-Pl^{pos} patients) and by fMLP★ (in iHSR-Tax^{pos} and iHSR-Pl^{pos} patients).

Supplementary References

Sref 1. Picard M, Pur L, Caiado J, Giavina-Bianchi P, Galvão VR, Berlin ST, Campos SM, Matulonis UA, Castells MC. Risk stratification and skin testing to guide re-exposure in taxane-induced hypersensitivity reactions. J Allergy Clin Immunol. 2016;137(4):1154-1164.

Sref 2. Ansotegui IJ, Melioli G, Canonica GW, Caraballo L, Villa E, Ebisawa M, et al. IgE allergy diagnostics and other relevant tests in allergy, a World Allergy Organization position paper. World Allergy Organ J. 2020;13(2):100080.

Sref 3. Regan JA, Cao Y, Dispenza MC, Ma S, Gordon LI, Petrich AM, Bochner BS. Ibrutinib, a Bruton's tyrosine kinase inhibitor used for treatment of lymphoproliferative disorders, eliminates both aeroallergen skin test and basophil activation test reactivity. J Allergy Clin Immunol. 2017 Sep;140(3):875-879.e1. doi: 10.1016/j.jaci.2017.03.013. Epub 2017 Apr 4. PMID: 28389390; PMCID: PMC7366359.

Sref 4. MacGlashan D Jr, Honigberg LA, Smith A, Buggy J, Schroeder JT. Inhibition of IgE-mediated secretion from human basophils with a highly selective Bruton's tyrosine kinase, Btk, inhibitor. Int Immunopharmacol. 2011 Apr;11(4):475-9. doi: 10.1016/j.intimp.2010.12.018. Epub 2011 Jan 14. PMID: 21238622; PMCID: PMC3345890.

Sref 5. Estupiñán HY, Berglöf A, Zain R, Smith CIE. Comparative Analysis of BTK Inhibitors and Mechanisms Underlying Adverse Effects. Front Cell Dev Biol. 2021 Mar 11;9:630942. doi: 10.3389/fcell.2021.630942. PMID: 33777941; PMCID: PMC7991787.
